# Air pollution is associated with the risk of neurodegenerative disorders: a prominent role of PM10

**DOI:** 10.1093/eurpub/ckac129.298

**Published:** 2022-10-25

**Authors:** A Gialluisi, S Costanzo, G Veronesi, G Zazzaro, A Cembalo, MM Ferrario, F Gianfagna, S Massari, L Iacoviello

**Affiliations:** 1 Department of Medicine and Surgery, University of Insubria, Varese, Italy; 2 Department of Epidemiology and Prevention, IRCCS NEUROMED, Pozzilli, Italy; 3 CIRA-Italian Aerospace Research Centre, Capua, Italy; 4 Mediterranea Cardiocentro, Naples, Italy; 5 Italian Workers’ Compensation Authority, INAIL, Rome, Italy

## Abstract

**Background:**

Studies revealed an implication of air pollution in neurodegenerative disorders, although this link remains unclear. Here, we investigated this testing multiple pollutants simultaneously.

**Methods:**

In the Moli-sani cohort (N = 24,325; ≥35 years; 51.9% women, baseline 2005-2010), we estimated yearly levels of exposure to nitrogen oxides (NOX, NO, NO2), ozone (O3), particulate matter (PM10) and BTX hydrocarbons (benzene, toluene and xylene) in 2006-2018, applying residence geo-localization of participants and Kriging interpolation algorithm to land measurements of air pollutants. We performed a principal component analysis and tested association of the resulting principal components (PCs) with the incident risk of Parkinson (PD) and Alzheimer disease (AD), through multivariable Cox PH regressions adjusted for age, sex and education level completed.

**Results:**

Over 24,308 subjects with pollution data available (51.9% women, 55.8(12.0) years), we extracted three PCs explaining ≥5% of pollution exposure variance: PC1 (38.2%, tagging PM10 exposure), PC2 (19.5%, O3/CO/SO2), PC3 (8.5%, NOx/BTX hydrocarbons). Over a mean follow-up of 10.9(2.1) years, we observed statistically significant associations of PC1 with an increased risk of PD (HR[CI] = 1.04[1.02-1.05]; 405 incident cases) and AD (1.06[1.04-1.08]; 218 cases). These associations were confirmed when we analyzed PM10 levels averaged over follow-up time, in models further adjusted for professional exposures like working class, compartment and toxic compounds and lifestyles like smoking and drinking habits, physical activity and adherence to Mediterranean diet (PD: 1.27 [1.19-1.37]; AD: 1.22[1.16-1.28] per 1 μg/m3 increase of PM10).

**Conclusions:**

This evidence supports an influence of air pollution - especially PM10 - on increased neurodegenerative risk in the Italian population, independent on concurring risk factors. This suggests reducing PM10 pollution as a potential strategy to reduce neurodegenerative risk.

**Key messages:**

• PM10 levels are associated with increased Parkinson and Alzheimer disease risk.

• This suggests to act on air pollution to reduce neurodegenerative risk in the general population.

